# Direct resin composite restoration of endodontically-treated permanent molars in adolescents: bite force and patient-specific finite element analysis

**DOI:** 10.1590/1678-7757-2019-0544

**Published:** 2020-04-27

**Authors:** Monise de Paula RODRIGUES, Priscilla Barbosa Ferreira SOARES, Márcio Alex Barros GOMES, Renata Afonso PEREIRA, Daranee TANTBIROJN, Antheunis VERSLUIS, Carlos Jose SOARES

**Affiliations:** 1 Universidade Federal de Uberlândia Faculdade de Odontologia Departamento de Dentística e Materiais Odontológicos UberlândiaMinas Gerais Brasil Universidade Federal de Uberlândia, Faculdade de Odontologia, Departamento de Dentística e Materiais Odontológicos, Uberlândia, Minas Gerais, Brasil.; 2 Universidade Federal de Uberlândia Faculdade de Odontologia Departamento de Periodontia e Implantologia UberlândiaMinas Gerais Brasil Universidade Federal de Uberlândia, Faculdade de Odontologia, Departamento de Periodontia e Implantologia, Uberlândia, Minas Gerais, Brasil.; 3 University of Tennessee Health Science Center College of Dentistry Department of Restorative Dentistry MemphisTennessee USA University of Tennessee Health Science Center, College of Dentistry, Department of Restorative Dentistry, Memphis, Tennessee, USA.; 4 University of Tennessee Health Science Center College of Dentistry Department of Memphis Tennessee USA Bioscience Research, University of Tennessee Health Science Center, College of Dentistry, Department of Memphis, Tennessee, USA.

**Keywords:** Finite element analysis, Patient specific modeling, Molar, Endodontically-treated teeth, Synthetic Resins, Bite force

## Abstract

**Objective:**

To evaluate the influence of three levels of dental structure loss on stress distribution and bite load in root canal-treated young molar teeth that were filled with bulk-fill resin composite, using finite element analysis (FEA) to predict clinical failure.

**Methodology:**

Three first mandibular molars with extensive caries lesions were selected in teenager patients. The habitual occlusion bite force was measured using gnathodynamometer before and after endodontic/restoration procedures. The recorded bite forces were used as input for patient-specific FEA models, generated from cone-beam computed tomographic (CT) scans of the teeth before and after treatment. Loads were simulated using the contact loading of the antagonist molars selected based on the CT scans and clinical evaluation. Pre and post treatment bite forces (N) in the 3 patients were 30.1/136.6, 34.3/133.4, and 47.9/124.1.

**Results:**

Bite force increased 260% (from 36.7±11.6 to 131.9±17.8 N) after endodontic and direct restoration. Before endodontic intervention, the stress concentration was located in coronal tooth structure; after rehabilitation, the stresses were located in root dentin, regardless of the level of tooth structure loss. The bite force used on molar teeth after pulp removal during endodontic treatment resulted in high stress concentrations in weakened tooth areas and at the furcation.

**Conclusion:**

Extensive caries negatively affected the bite force. After pulp removal and endodontic treatment, stress and strain concentrations were higher in the weakened dental structure. Root canal treatment associated with direct resin composite restorative procedure could restore the stress-strain conditions in permanent young molar teeth.

## Introduction

First permanent molar teeth exert the highest force during chewing.^1^ Masticatory performance is usually assessed by masticatory tests and bite force measurement.^2^ Any structural alteration affecting tooth integrity may impact the masticatory process.^3^ Permanent first molars are the first posterior teeth to erupt and the most affected by dental caries.^4^ The deep sulcus and fissures on the occlusal surface facilitate accumulation of acid produced by bacteria, as well as the early eruption contributes further to caries progression.^4^ Progression in dental caries can result in pulp injuries, periapical infection and tooth loss.^5^ Damage caused by the caries process can be so extensive that the loss of structural integrity can affect masticatory functions. Dental caries can negatively impact adolescents, resulting in uncomfortableness, pain, functional impact, difficulty in chewing, and emotional problems.^6^ Further progression of caries may result in premature tooth loss with serious consequences, including a collapse of the posterior dental arch space and, consequently, the extrusion of antagonist tooth, interfering in occlusal plane.^7^ Therefore, management of carious permanent molars is important for the quality of life and growing process of children and adolescents.^8^

Physiological bite forces have an important role on the progression of carious lesions to cavitation.^9^ Bite loading applied over multiple cycles can cause and propagate stresses, contributing to the failures clinically observed.^10^ Pulpal involvement caused by extensive caries may result in pain and reduce mastication force due to natural adaptation. When endodontic treatment is recommended, root canal access and pulp removal can eliminate the pain in the first section; however, without recovering the resistance of weakened tooth structure. Performing endodontic treatment followed by direct restoration with bulk-fill resin composite is a good alternative to maintain tooth function.^11^ Resin composites are considered the first choice for restoration of severely damaged teeth in young patients due to their esthetic quality, conservative approach, and handling characteristics.^12 , 13^ Bulk-fill represents a recent innovation to simplify resin composite insertion, which may also decrease polymerization shrinkage stresses and cuspal deflection.^14 , 15^

Biomechanical analysis of endodontically-treated molar teeth with extensive loss of tooth structures caused by extensive caries in adolescents is scarce. How restorative treatments improve the bite force and promote homogeneous stress distribution have not yet been determined. Moreover, large areas of unsupported enamel resulted from dentin destruction could increase the occurrence of tooth fracture under functional bite forces.^16^ In addition, functional bite after pulp removal is lost, and the normal load applied on a weakened molar can result in tooth fracture. Our study sought to evaluate how different levels of tooth structure loss in endodontically-treated permanent molars restored with direct bulk-fill resin composite affect stress distribution and bite force in three adolescent patients. The stress was evaluated using patient-specific finite element models that represent three progressive structure losses of molar teeth, which models created from each individual patient’s anatomy, allowing a loading that more truthfully mimics individual conditions.^17^ The null hypothesis was that the root canal treatment followed by direct bulk-fill resin composite restorative process would not recover the bite force and would not improve the stress distribution of a permanent molar.

## Methodology

### Patient selection

**Figure 1:**
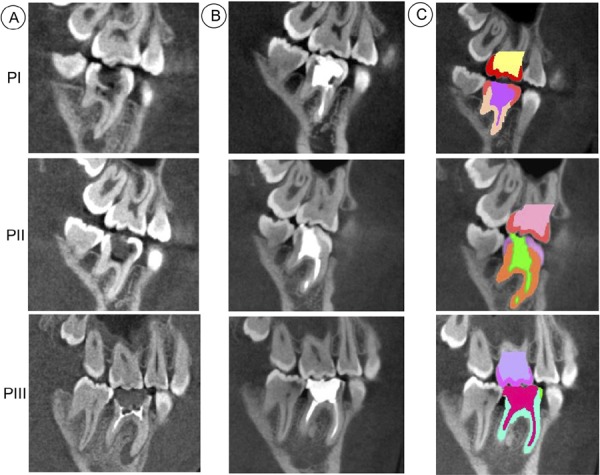
Cone-beam computed tomographic images from the three patients: A. the initial conditions of severe tooth structure loss; B. after endodontic treatment and direct resin composite restoration; C. segmentation using Mimics software. PI- both marginal ridges and all cusps remaining; PII- one marginal ridge remaining and one lingual cusp lost; and PIII- loss of both marginal ridges and lingual cusps, only the buccal cusps remaining

**Figure 2:**
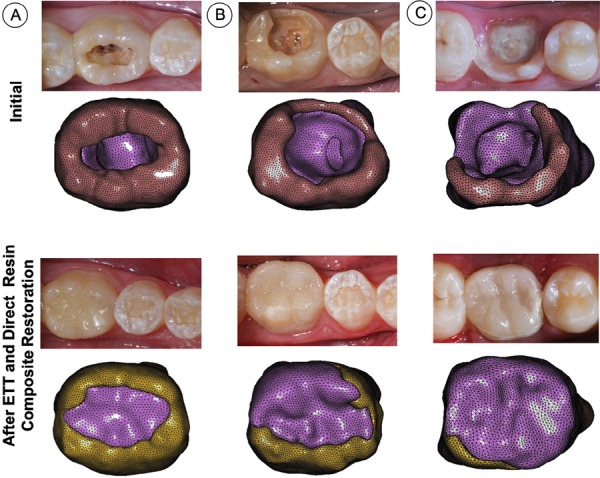
Clinical pictures (after endodontic treatment) and corresponding patient-specific finite element models. A, PI, both marginal ridges and all cusps remaining; B, PII, one marginal ridge remaining and loss of one lingual cusp; and C, PIII, loss of both marginal ridges and lingual cusps, only the buccal cusps remaining

This study was approved by the ethics committee (protocol nº 1.685.725). Three patients were selected (9, 10 and 12 years old, hereinafter “adolescents”) with a first lower permanent molar affected by caries with pulpal involvement resulting in dental pain requiring endodontic treatment, considered as biopulpectomy. The patients selected had teeth with various and progressive levels of tooth structure loss ( Figures 1 and 2 ): PI, both marginal ridge and all cusps remaining ( Figure 2A ); PII, one marginal ridge remaining and one cusp (lingual) lost ( Figure 2B ); and PIII, both marginal ridges remaining and both lingual cusps lost (buccal cusps remaining) ( Figure 2C ).

### Intraoral bite force measurement

The maximum bite forces (Newtons) each patient could perform were measured at initial condition (before endodontic and restorative procedures) and postoperatively, using a mini load cell (Gnatodinamômetro Digital Especial; Kratos, Cotia, SP, Brazil) with a 100 Kgf (1000 N) capacity and 5 N accuracy. The dynamometer contains a fork attached with two 6-mm thick slides each and 3.0 mm of space between then. The patients remained seated in a dental chair, with their heads in a comfortable position and the Frankfurt horizontal plane parallel to the floor with maximum oral opening. Polytene platforms with 8.0 mm in diameter connected to each fork of the dynamometer was positioned on the center of the occlusal surface of the first maxillary molar. The device touched only the target maxillary molar involved in the study. The measurements were repeated five times with a 3-second interval between each bite for recording of occlusal bite force. The adolescents were instructed to bite as hard as possible on the gauge in the region of primary molars without moving the head. Each patient’s mean bite was used for the load application in the finite element analysis.

### Cone-beam image acquisition

Before and after the endodontic treatment and restorative procedure, the teeth and surrounding support structures were imaged using cone-beam computed tomographic scanning (i-CAT GXCB-500; Imaging Sciences International, Hatfield, Pennsylvania) with the median sagittal plane perpendicular to the horizontal plane and the occlusal plane parallel to the horizontal plane. Voxel dimensions were 0.125 mm ( Figure 1A, 1B and 1C ). In total, 704 slices were obtained with 6 seconds of acquisition and exposure parameters of 120 kV and 5.0 mA. The projection data of the first permanent molar and support structures were exported using Digital Imaging and Communication in Medicine (Dicom) file format.

### Endodontic and restorative procedures

The cavity preparations were limited to removal of caries infected tissue. After caries removal, for the endodontic procedure, root canals were shaped by rotary instruments (ProTaper Next, Dentsply Malleifer, Petrópolis, RJ, Brazil) following the crown-down technique. The rotational speed and torque were set at 300 rpm and 2 Ncm, respectively, according to the manufacturer’s recommendations. Throughout the preparation process, the root canals were irrigated with 2.5% sodium hypochlorite (Chlorine Rio, São José do Rio Preto, SP, Brazil) using a Luer Lock 5 mL syringe (BD, Curitiba, PR, Brazil) and NavTip (Ultradent, South Jordan, UT, USA). The final irrigation was performed with 17% EDTA solution and physiological saline solution to remove the remaining debris. A calcium hydroxide-based intracanal medication was used between appointments. The instrumented root canals were obturated using the lateral condensation technique with a pre-selected gutta-percha master cone ProTaper Next (Dentsply Malleifer), conventional gutta-percha accessory cones FM (Dentsply Malleifer), and calcium hydroxide-based cement (Sealer 26, Dentsply, São Paulo, SP, Brazil). Excess gutta-percha was removed from the pulp chamber by pre-warmed Paiva’s compactors and cleaned with isopropyl alcohol.

The restorative procedure was performed on the same visit when the endodontic treatment was concluded. The remaining enamel was etched with 37% phosphoric acid (Adper Scotchbond Etching, 3M ESPE, St. Paul, USA) for 30 seconds and rinsed with water. An adhesive system (Single Bond Universal, 3M ESPE) was applied actively in two layers, and light cured using an LED light curing unit with 1200 mW/cm^2^ (Optilight Max, Gnatus, Ribeirão Preto, SP, Brazil) for 20 seconds. The teeth were restored using a bulk-fill regular paste resin composite (Filtek Bulk Fill Posterior, 3M ESPE) inserted in a 5-mm thick increment to fill the pulp chamber and restore the missing dentin structure. A second increment of 4 to 5 mm of thickness was used for restoring the occlusal area. Each increment was light-cured for 20 seconds. The bite force was measured five times with a 3-second interval 24 h after restorative procedure following the previously described methodology. The restored molars were followed for 2 years after treatment following these criteria: marginal integrity/adaptation, marginal discoloration, recurrent caries, retention of resin composite restorations and postoperative sensitivity.

### Finite element analysis

**Figure 3:**
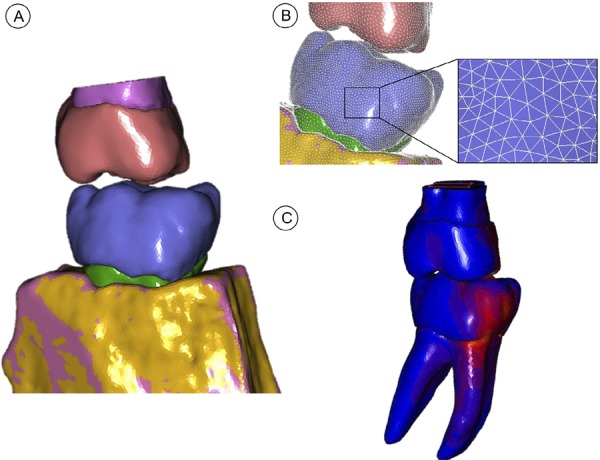
A schematic showing the approach for developing a patient-specific finite element model. A, 3D reconstruction of the dental structure using Mimics and 3-Matic software. B, the final mesh created with Mimics, 3-Matic, and Patran software. C, nonlinear analysis simulating the biting force with clinically obtained inputs using Marc/Mentat software

A three-dimensional (3D) reconstruction was created based on the cone-beam tomography images (i-CAT GXCB-500™ Imaging Sciences International, Hatfield, Pennsylvania). The images were exported in Digital Imaging and Communication in Medicine (DICOM) file format and imported into an interactive medical imaging software (Mimics 18.0, Materialise Dental, Leuven, Belgium). The segmentation of the tooth structures and restorative materials was accomplished using image density thresholding.^18 , 19^ The gutta-percha was not modeled because of the very low elastic modulus that would not impair the stress distributions. Periodontal ligament layers (0.2 mm thick) were imposed on tooth roots by Boolean operations. After segmentation, the 3D triangle-based surface of each tooth structure was exported in Stereo Lithography (STL) format ( Figure 3A ).

The STL surface models were imported and meshed in MSC.Patran^®^ 2010 (MSC.Software, MSC software, Santa Ana, CA, USA) with tetrahedral elements, which is element number 134 ( Figure 3B ). The created volumetric element mesh was imported in a FEA software package (MSC.Marc/Mentat; MSC.Software) to perform the structural analysis ( Figure 3C ). A mesh was generated with a 0.5 mm element size subjected to convergence analysis before mechanical simulation. All materials were considered linear-elastic, isotropic, and homogeneous. The applied material properties (elastic modulus, Poisson’s ratio, tensile strength, and compressive strength) were obtained from the literature ( Table 1 ). The carious tissue properties were not simulated. Interfaces between model components were prescribed as bonded contacts, preventing relative motion along model interfaces. Nodes on the mesial and distal sectioned surfaces of the bone structure were rigidly fixed in all directions.

**Table 1:** Material and tissue properties

Materials/ Structures	Elastic Modulus (MPa)	Poisson’s ratio	Tensile Strength (MPa)	Compressive Strength (MPa)	References
Dentin	18600	0.31	98.7	297.0	Vianna et al., 2018^36^
Filtek Bulk Fill Posterior resin composite	12800	0.24	42	169.0	Oliveira et al., 2016^37^
Periodontal Ligament	50	0.45	-	-	Vianna et al., 2018^36^
Cortical Bone	13700	0.30	-	-	Vianna et al., 2018^36^
Cancellous Bone	1370	0.30	-	-	Vianna et al., 2018^36^
Enamel	84100	0.30	10.3	384.0	Vianna et al., 2018^36^

The individual bite force means (N) determined clinically before and after endodontic and restorative procedures were used as input loading. Occlusal bite force means from each patient were used as an input load parameter for the pre-processing computer simulation using the finite element method. The force was applied by placing a vertical force on the control surface of the antagonist. During the analysis, contact status determined that the antagonist teeth contacted the mandibular molars according to the positioning acquired by the computed tomographic image. Three loading conditions were simulated in each patient: M1, initial condition of initial bite force on the original tooth (before endodontic and restorative treatment); M2, postoperative (final) bite force applied to the model of the original tooth; and M3, final bite force on the tooth after endodontic and restorative procedures. Stress distributions were analyzed using modified von Mises equivalent stresses to account for the difference between compressive and tensile strengths. The tooth structure maximum principal strain (µS) was recorded during load increments for all models and plot for comparison among the three conditions for all models.

## Results

### Intraoral bite force measurement

The mean and standard deviation of bite forces (N) measured at the initial unrestored condition and at the final condition after endodontic and restorative procedures were: PI, 30.1±5.9 and 136.6±16.6; PII, 34.3±10.2 and 133.4±21.4; PIII, 47.9± 12.0 and 124.1±17.5, respectively. The mean bite force of the three patients increased 260% (36.7±11.6 to 131.9±17.8). The bite forces were significantly lower in the presence of extensive caries with pulp involvement, regardless of the level of tooth structure loss. After the endodontic treatment and resin composite restoration, the bite force increased considerably. The clinical parameters of color match, surface quality, marginal integrity/adaptation and marginal discoloration were evaluated, and the restorations performed adequately after a 2-year follow-up period.

### Stress and strain distribution

**Figure 4:**
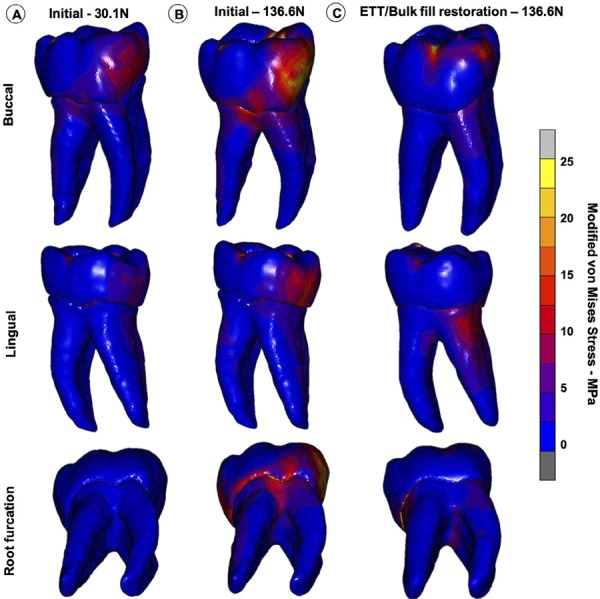
Modified von Mises stress distributions of three loading conditions in PI, (both marginal ridge and all cusps remaining). A, M1 – loading the unrestored tooth with the initial bite force (30.1 N). B, M2 – loading the unrestored tooth with the final bite force (136.6 N) C, M3 – loading the endodontically-treated and restored tooth with the final bite force (136.6 N)

**Figure 5:**
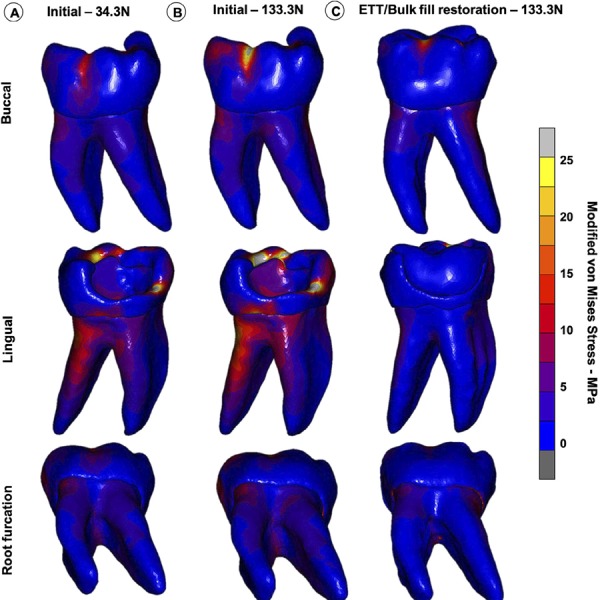
Modified von Mises stress distributions of three loading conditions in PII, (both marginal ridge and all cusps remaining). A, M1 – loading the unrestored tooth with the initial bite force (30.1 N). B, M2 – loading the unrestored tooth with the final bite force (136.6 N) C, M3 – loading the endodontically-treated and restored tooth with the final bite force (136.6 N)

**Figure 6:**
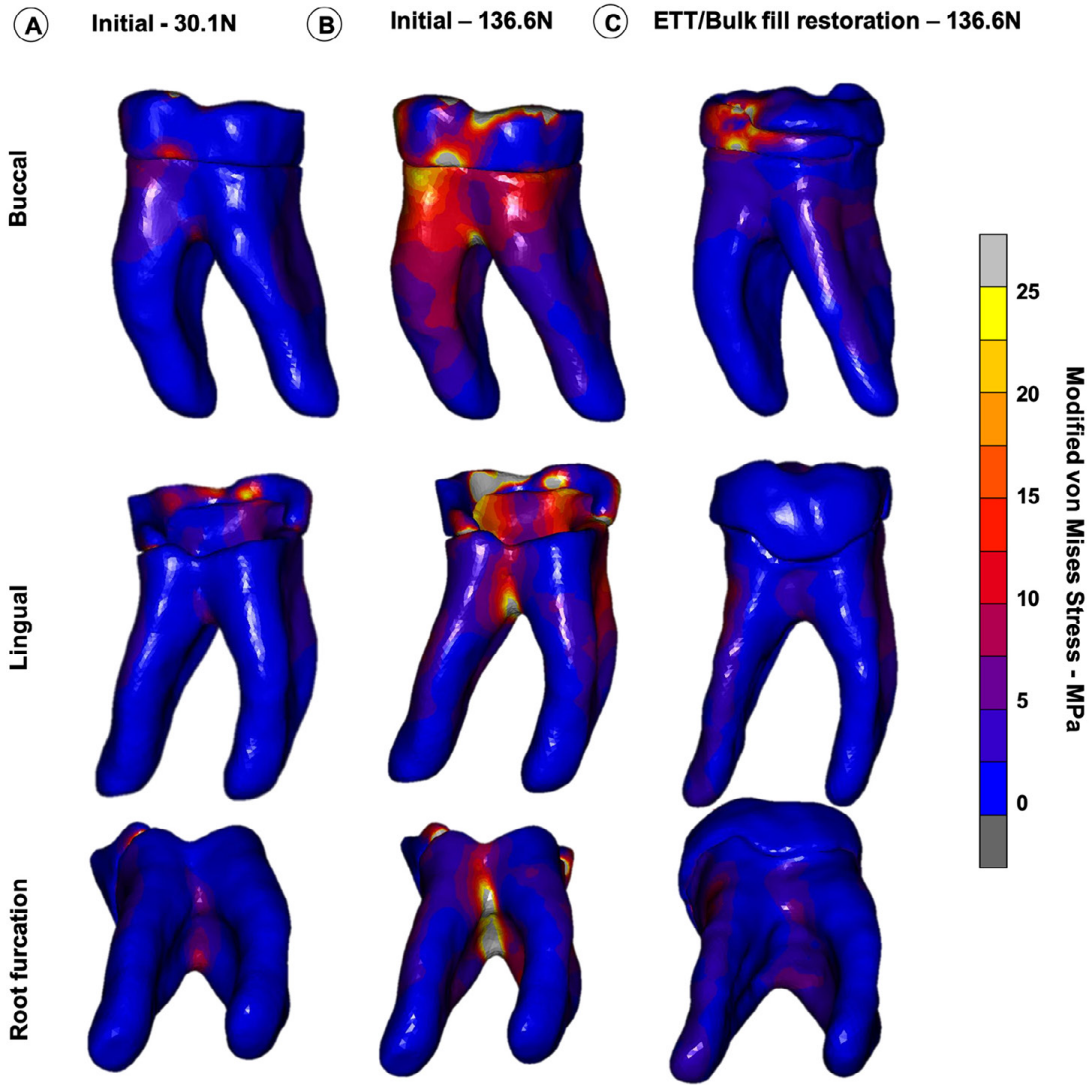
Modified von Mises stress distributions of three loading conditions in PIII, (both marginal ridge and all cusps remaining). A, M1 – loading the unrestored tooth with the initial bite force (30.1 N). B, M2 – loading the unrestored tooth with the final bite force (136.6 N) C, M3 – loading the endodontically-treated and restored tooth with the final bite force (136.6 N)

**Figure 7:**
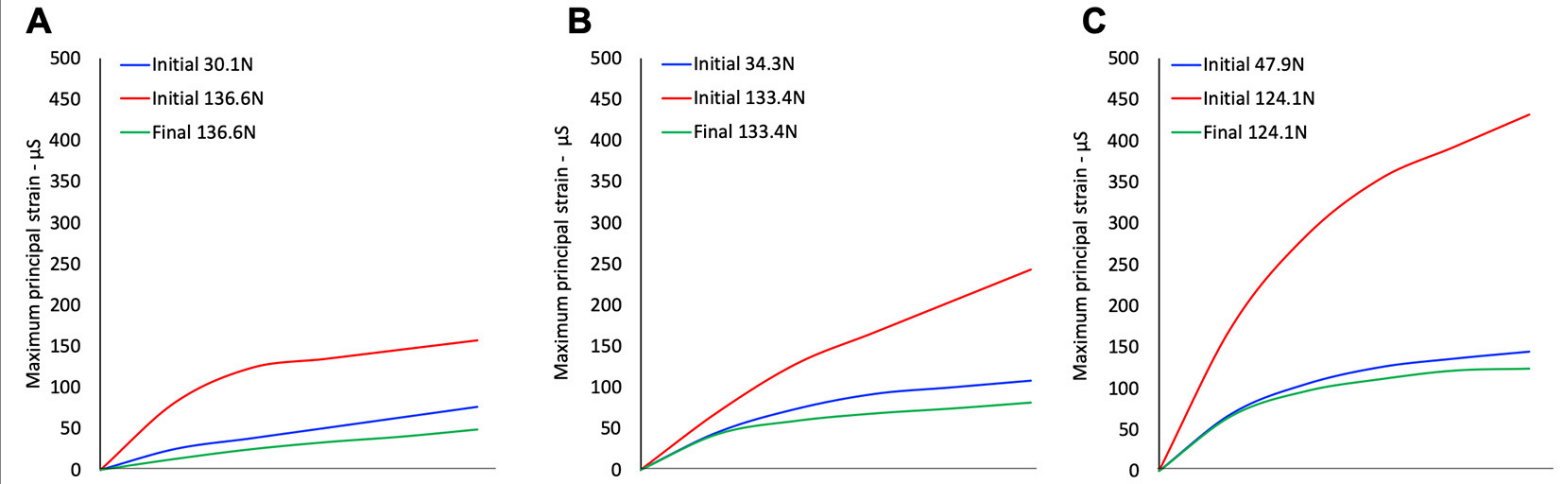
Maximum principal strain curves during loading increments of three loading conditions M1 – loading the unrestored tooth with the initial bite force, M2 – loading the unrestored tooth with the final bite force and M3 – loading the endodontically-treated and restored tooth with the final bite force in A, PI. B, PII. And C, PIII (both marginal ridge and all cusps remaining)

The modified von Mises stress distribution for the tooth structure at the initial unrestored condition and after resin composite restoration are shown for the three teeth in Figures 4 , 5 and 6 . For the M1 loading condition, in which the lower patient adaptive bite force was simulated, the stresses were significantly lower on the remaining coronal tooth structure ( Figures 4A , 5A and 6A ). The M2 loading condition, in which the final (high) bite force was simulated on the unrestored tooth ( Figures 4B , 5B and 6B ), the stress concentrations were significantly higher than for the M3 loading condition, in which the final bite force was placed on the endodontically-treated and restored tooth ( Figures 4C , 5C and 6C ). High stress concentrations were observed on the tooth structure remains and at the root furcation of the molar ( Figures 4B , 5B and 6B ) for the M2 condition. After the endodontic and restorative procedure, the stresses were more evenly distributed over the root and considerably reduced at the root furcation ( Figures 4C , 5C and 6C ). The tooth structure strain curves (µS) recorded during load increments for all models and plot for comparison among the three conditions for all models are shown in Figure 7 . The strain was always higher for the M2 loading condition. The bite force adaptation determined lower tooth structure strain. After restorative procedure, the tooth structure strain reduced significantly.

## Discussion

Three different levels of the dental structure loss caused by caries and pulp involvement on first permanent mandibular molars were simulated to study the influence of the amount of remaining dental tissue on the bite force and stress distributions in mandibular molars. The amount of the dental structure loss had no effect on the bite force when comparing the initial conditions, demonstrating that the condition of the pulp may be the main factor for determining bite force adaptation.^22^ Although this description can be interpreted as a speculative thesis for supporting bite force adaptation, it was very clear during the initial bite force measurement, when all patients reported that they could not apply more force because they are feeling pain and concerned about the tooth fracture. However, a large difference was observed in bite force magnitude and stress distribution before and after endodontic treatment and resin composite restoration. Therefore, the null hypothesis was rejected.

Bite force has been widely used as an acceptable test for masticatory system function.^17 , 23^ The bite forces measured at initial condition for the three patients were substantially lower than after the endodontic and restorative procedures. The lower bite force could have been caused by chewing alteration due to pulp pain.^20^ Dental pathologies, such as poor periodontal and dental conditions, can result in a reduced bite force.^24^ A reflex withdrawal reaction to the pain evoked on biting and the induction of masseteric inhibitory periods^25^ can explain the lower bite forces.^22^ Loss of dental hard tissues due to extensive dental caries generated smaller occlusal contact areas.^24 , 25^ Moreover, the weakened coronal tooth structure may determine natural adaptation by reducing the bite force to prevent tooth fracture.^28^ Patients with extensive caries and/or pulpal involvement usually lose dental function and have difficulty in eating, which should be considered an indicator of oral problems and may negatively impact quality of life. The increase in bite force after endodontic treatment and resin composite restoration resulted in similar bite forces for all patients, regardless of differences in amount of lost dental structure. Pain removal and adequate coronal reconstruction by resin composite restorative procedure resulted in an increase of about 260% in bite force. This may have direct impact on an improved nutritional development of young patients.^29^

Advanced stages of carious lesions in posterior teeth have been associated with a negatively impacted quality of life.^30^ Patient-specific FEA allowed investigation of stress distributions in specific severely damaged teeth, providing insights that cannot be obtained through clinical examination, but also may not be extrapolated for all different clinical conditions. Computed tomographic scanned data allowed efficient generation of specific three-dimensional models.^17 , 31 , 32^ The limited number of the models, only one *per* group, may be considered a limitation of this study. However, it is important to emphasize that the finite element analyses have used only one general model to draw important conclusions. Combining 3D FEA with the specific bite force can further improve the prediction for fracture risks in individual tooth. When the final bite force was simulated on weakened teeth, high stress concentrations were found at the root furcation and at the remaining coronal structure, indicating an increased risk of tooth fracture.^31^ Greater tooth structure loss resulted in higher stress concentrations when comparing the three levels of the dental structure loss. This highlights the need for early intervention regardless of pulpal involvement. This study also emphasized the importance for adequate provisional and early definitive resin composite restoration after starting endodontic intervention, since the patient is more comfortable in mastication when dental pain is removed, resulting in higher bite forces, thus increasing the possibility of tooth fracture.

After the endodontic treatment and bulk-fill resin composite restoration, the stress and strain were significantly reduced in weakened areas, being better distributed in the root areas with more volume of the dentin tissue. The remaining unsupported enamel and the furcation area on root dentin are local with higher stress and strain concentration for the models without restoration that received final load. These areas can represent the location of tooth fracture initiation, which is more reasonable when compared with frequent tooth fractures clinically observed. Based on the results of our study, the teeth with more preserved tooth structure also had more evenly distributed stress after restoration, thus reducing the failure risks. Therefore, preserving more coronal tooth structure leads to better stress distributions and consequent reduced tooth fracture risks. Clinicians should therefore try to maintain as much coronal structure as possible during root canal treatment and restorative procedures. For example, using the pulp chamber for retention of the coronal reconstruction.^29^ After endodontic treatment and bulk fill resin composite restoration, the strain is very similar for the three models, demonstrating the biomechanical recovering capacity of the restorative protocol. The follow up of 2 years of these 3 clinical conditions used as a reference for finite element simulation demonstrated good clinical performance of bulk-fill composite resin to restore large cavities in endodontically-treated molar teeth. The option for bulk-fill composite resin for restoring endodontically-treated molar teeth is based on the good clinical performance described in recent systematic reviews and metanalysis when compared with incremental filling technique.^34 , 35^ Other important aspect for adolescents is that restorations in molar teeth using regular paste bulk-fill resin composites require a shorter time to be performed than using the incremental filling technique.^36^ This study showed, based on biomechanics, that bulk-fill resin composite restoration following endodontic treatment is a good strategy for maintaining molar teeth that have been severely affected by caries. Bulk-fill resin composite, besides presenting lower polymerization shrinkage stresses and cuspal deflection than conventional composite resin,^11 , 37^ was shown to be able to provide relatively even distribution of stresses in the remaining tooth structure, as well as improved masticatory ability in young patients.

## Conclusions

Extensive caries in molar teeth in the specific clinical conditions simulated in this study negatively affected bite force and caused higher stress concentrations in the weakened tooth structure. Early definitive resin composite restoration should be performed after endodontic intervention, since higher bite forces are achieved when dental pain is removed, thus increasing the possibility of tooth fracture. Endodontic treatment in molar teeth followed by direct resin composite restoration was an effective method to reestablish oral biomechanical performance in these three adolescents.
